# Identification and Pharmacokinetics of Multiple Potential Bioactive Constituents after Oral Administration of Radix Astragali on Cyclophosphamide-Induced Immunosuppression in Balb/c Mice

**DOI:** 10.3390/ijms16035047

**Published:** 2015-03-05

**Authors:** Menghua Liu, Panlin Li, Xuan Zeng, Huanxian Wu, Weiwei Su, Jingyu He

**Affiliations:** 1 Guangdong Provincial Key Laboratory of New Drug Screening, School of Pharmaceutical Sciences, Southern Medical University, Guangzhou 510515, China; E-Mail: wuhuanxian37@163.com; 2 State Key Laboratory of Organ Failure Research, Guangdong Provincial Institute of Nephrology, Southern Medical University, Guangzhou 510515, China; 3 Guangzhou Quality R&D Center of Traditional Chinese Medicine, Guangdong Key Laboratory of Plant Resources, School of Life Sciences, Sun Yat-Sen University, Guangzhou 510275, China; E-Mails: lipanlin@gmail.com (P.L.); zengx6@mail2.sysu.edu.cn (X.Z.); lssswwhk@gmail.com (W.S.); 4 Guangzhou Institute of Advanced Technology, Chinese Academy of Sciences, Guangzhou 511458, Guangdong, China

**Keywords:** Radix Astragali, serum pharmacochemistry, absorbed components, pharmacokinetics

## Abstract

Radix Astragali (RA) is one of the commonly-used traditional Chinese medicines (TCMs) with an immunomodulatory effect confirmed in the clinic. In order to better understand the material basis for the therapeutic effects, this study was to investigate the absorbed components and their pharmacokinetic profile after oral administration of RA on cyclophosphamide-induced immunosuppression in Balb/c mice. As a result, 51 compounds in RA extract and 31 prototype compounds with nine metabolites were detected in mice plasma by the ultra-fast liquid chromatography (UFLC)-DAD-Q-TOF-MS/MS method. The pharmacokinetic parameters of five main constituents, including calycosin-7-*O*-glucoside, ononin, calycosin, formononetin and astragaloside IV, were obtained using HPLC-MS/MS. These results offered useful information for research on the pharmacological mechanism of RA and for its further development.

## 1. Introduction

Immunosuppression is a kind of common clinical manifestation during chemotherapy. Since its forms are nonspecific and lead to diminution of response to a variety of agents, the state of immunologic unresponsiveness may have undesirable features, for instance placing the host at risk of infection by a variety of opportunistic agents, subsequently increasing the difficulty of the treatment of the illness [1,2]. Therefore, alleviating immunosuppression is one of the important approaches in chemotherapy.

Radix Astragali (RA) was recorded originally in “Shen Nong Ben Cao Jing”. RA, described as the root of *Astragalus membranaceus* (Fisch.) Bge. var. mongholicus (Bge.) Hsiao or *Astragalus membranaceus* (Fisch.) Bge., is one of the commonly-used traditional Chinese medicines (TCMs) in Asia, such as China, Japan and Korea [3,4,5]. Pharmacological research showed that it has anti-inflammatory [6,7], anti-oxidative [8], hepatoprotective [9], kidney-protective [10] and neuro-protective activities [11]. Of even greater concern is the immunomodulatory effect of RA in the clinic when it was used as either a single herb or as a formula in combination with other Chinese medicines. Modern studies confirmed that its immunomodulatory effect is closely related to the protective effect on immune organs and the regulative effect on immune cells, including lymphocytes, leukocytes and macrophagocytes [12,13,14,15,16]. In addition, the immunomodulatory effect of RA in immunosuppressed mice induced by cyclophosphamide treatment was tested in our previous study. Compared with the model group, the RA-treated group (6 g/kg body weight) had accelerated recovery of the spleen index, peripheral white blood cell and bone marrow cell counts and enhanced splenic nature killer cell activity and peritoneal macrophage phagocytosis. Meanwhile, the levels of interferon-γ (IFN-γ), interleukin-12P70 (IL-12P70), IL-6 and IL-17 were restored, obviously (data to be published). These results showed that RA had an immune-enhancement effect on cyclophosphamide-induced immunosuppression in Balb/c mice, which was consistent with that of the reports [12,13,14,15,16].

It is generally known that chemical compounds are responsible for the therapeutic effect of medicinal plants. Clarifying which constituents from herbs have a therapeutic effect is very important for Chinese medicines, to understand their pharmacological mechanism and clinical application better. Although more than 100 compounds, such as flavonoids, saponins, polysaccharides and amino acids, have so far been isolated and identified in AR [17,18,19], little has been known about which constituents absorbed into blood produce the immune-enhancement effect in pathological conditions after oral administration of RA, till now.

Therefore, the aims of this study are three: (1) to profile the absorbed constituents after oral administration of RA in cyclophosphamide-induced immunosuppressive Balb/c mice; (2) to discover the metabolites in blood as much as possible; and (3) to describe the pharmacokinetics of the main compounds in immunosuppressive mice.

## 2. Results and Discussion

### 2.1. Identification of Compounds in RA Extract

**Figure 1:**
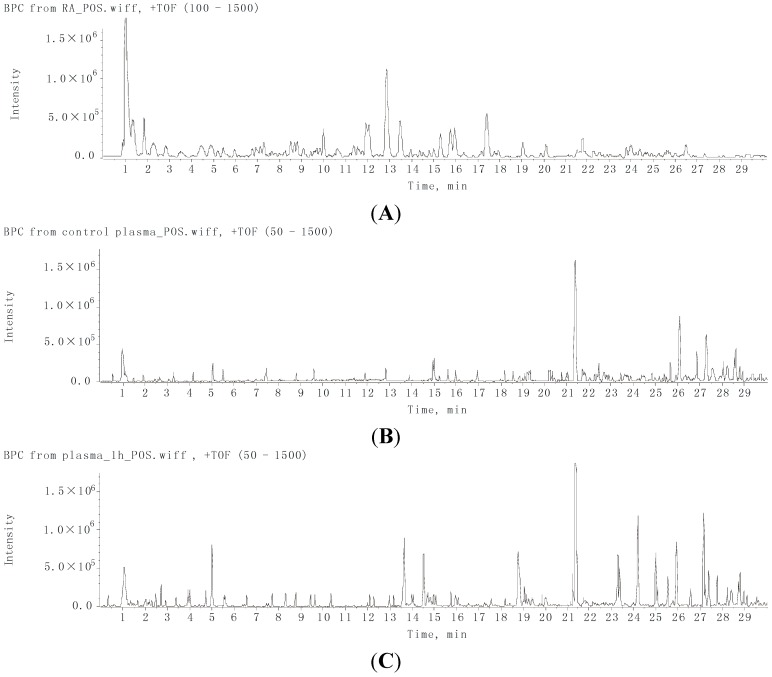
The positive ion chromatograms of RA extract (**A**); control plasma (**B**) and dosed plasma at 1 h (**C**).

**Figure 2:**
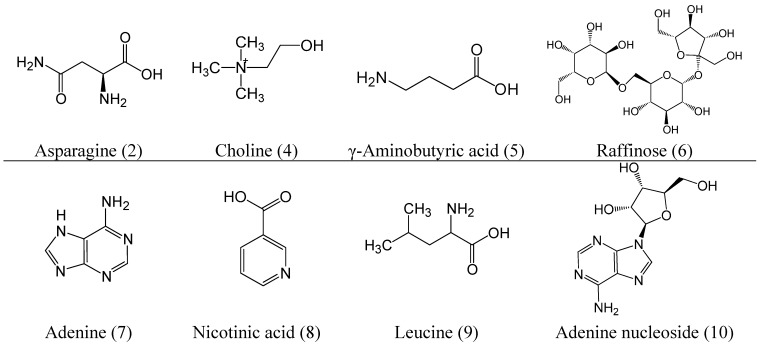

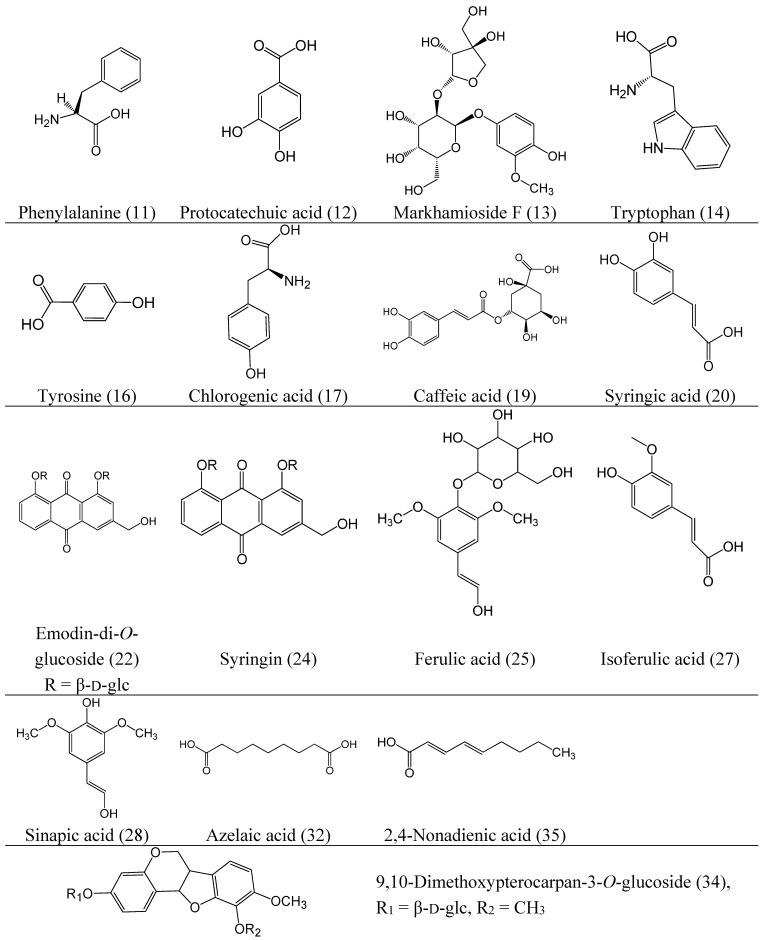

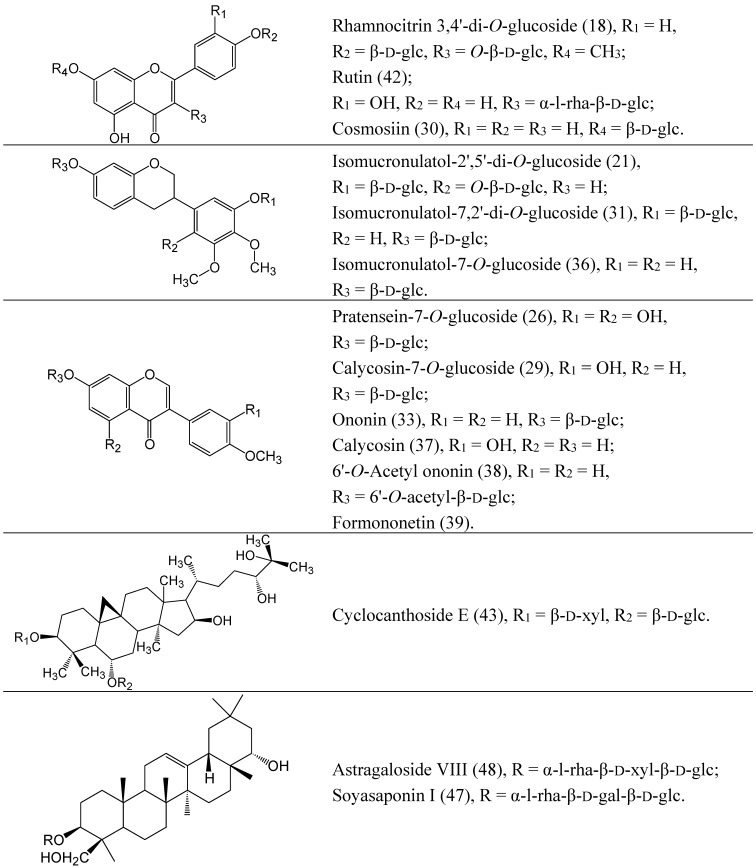

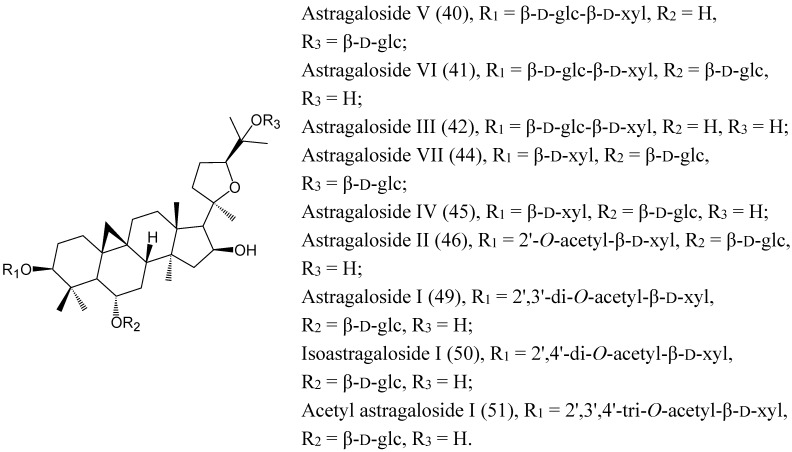
Chemical structures of the compounds identified in RA extract and mice plasma.

As shown in Table 1 and Figure 1, a total of 51 compounds in RA extract, including amino acids, organic acids, flavonoids and saponins, were identified or tentatively characterized based on their LC retention time, DAD data, accurate molecular weight and fragmentation behaviors, both in positive and negative mode. Their structures are shown in Figure 2.

All compounds, except Peaks 15, 24, 25, 27, 28 and 39, have been reported in our previous studies [18,20]. By comparing the individual peak chromatographic behavior with those of reference standards, Peaks 15, 24 and 39 were unequivocally confirmed as hydroxylbenzoic acid, syringin and formononetin, respectively. Peaks 25 and 27 were a pair of isomers. Peak 25 showed a quasi-molecular ion at *m*/*z* 193.0510 ([C_10_H_9_O_4_]^−^, calcd. 193.0459), which generated fragment ions at *m*/*z* 178.0257 ([C_9_H_6_O_4_]^−^, calcd. 178.0260) and 149.0611 ([C_9_H_9_O_2_]^−^, calcd. 149.0597) by the losses of the CH_3_ and CO_2_ group, respectively, and *m*/*z* 134.0380 ([C_8_H_6_O_2_]^−^, calcd. 134.0362) is the fragment ion due to simultaneously losing the CH_3_ and CO_2_ groups from *m*/*z* 193.0510. Peak 25 was unequivocally identified as ferulic acid. Peak 27, with its retention time at 11.90 min, generated the same fragment ions as those of Peak 25. According to the literature [21], Peak 27 was tentatively assigned as isoferulic acid. Peak 28 displayed an [M + H]^+^ ion at *m*/*z* 225.0759 ([C_11_H_13_O_5_]^+^, calcd. 225.0757) and yielded ions at *m*/*z* 207.0648 ([C_11_H_11_O_4_]^+^, calcd. 207.0651), 179.0671 ([C_10_H_11_O_3_]^+^, calcd. 179.0702) and 175.0409 ([C_10_H_7_O_3_]^+^, calcd. 175.0390) by the losses of H_2_O, HCOOH and H_2_O + CH_3_OH, groups, respectively. The negative ion ESI-MS spectra showed [M − H]^−^ ion at *m*/*z* 223.0617 ([C_11_H_11_O_5_]^−^, calcd. 223.0601). The product ions of 205.0514 ([C_11_H_9_O_4_]^−^, calcd. 205.0495), 179.0710 ([C_10_H_11_O_3_]^+^, calcd. 179.0702) and 163.0401 ([C_9_H_7_O_3_]^+^, calcd. 163.0390) indicated the presence of hydroxyl, carboxyl and methoxyl groups. Thus, Peak 28 was tentatively identified as sinapic acid, but its structure needs to be further confirmed by the NMR method.

**Table 1:** LC-DAD-TOF-MS/MS data for the identification of multiple constituents in Radix Astragali (RA) extract, absorbed compounds and metabolites in immunosuppressed mice plasma.

No.	T_R_ (min)	Formula	[M + H]^+^ (Error, ppm)	[M − H]^−^ (Error, ppm)	Fragment Ions in Positive (+) Ion Mode	Fragment Ions in Negative (−) Ion Mode	Identification	Source		
								RA	Control Plasma	Dosed Plasma
1	0.96	C_6_H_12_O_6_		179.0563 (+0.79)	203.0521 [M + Na]^+^ (−2.4), 143.0327 [M + Na − C_2_H_4_O_2_]^+^	-	Hexose	+	+	0.25–36 h
2	0.97	C_4_H_8_N_2_O_3_	133.0607 (−0.7)	131.0472 (+7.6)	-	114.0194 [M − H − NH_3_]^−^, 113.0354 [M − H − H_2_O]^−^, 95.0257 [M − H − CH_2_O_2_]^−^	Asparagine ^a^	+	+	1–36 h
3	1.00	C_12_H_22_O_11_	-	341.1089 (−0.08)	365.1053 [M + Na]^+^ (−0.36), 203.0532 [M + Na − glc]^+^, 185.0425 [M + Na − C_6_H_12_O_6_]^+^	179.0553 [M − H − glc]^−^	Disaccharide	+	-	0.25–4 h
4	1.02	C_5_H_13_NO	104.1071 (1.0)	-	60.0833 [M + H − C_2_H_5_O]^+^	-	Choline ^a^	+	+	0.25–36 h
5	1.05	C_4_H_9_NO_2_	104.0706 (−0.1)	102.0615 (+1.0)	-	-	γ-Aminobutyric acid ^a^	+	+	0.25–36 h
6	1.12	C_18_H_32_O_16_	-	503.1621 (+1.7)	527.1583 [M + Na]^+^ (+0.1)	161.0453 [M + H − 2glc]^−^	Raffinose ^a^	+	-	0.25–3 h
7	1.21	C_5_H_5_N_5_	136.0617 (−0.6)	134.0473 (+1.0)	119.0351 [M + H − NH_3_]^+^, 92.0248 [M + H − NH_3_ − HCN]^+^	107.0366 [M + H − HCN]^+^, 72.0186	Adenine ^a^	+	-	-
8	1.37	C_6_H_5_NO_2_	124.0393 (−0)		106.0285 [M + H − H_2_O]^+^, 80.0500 [M + H − CO_2_]^+^, 78.0343, 53.0417	-	Nicotinic acid ^a^	+		0.25–4 h
9	1.63	C_6_H_13_NO_2_	132.1018 (−1.0)	130.0874 (+1.0)	86.0975 [M + H − CH_2_O_2_]^+^, 69.0714 [M + H−NH_3_ − CH_2_O_2_]^+^	-	Leucine ^a^	+	+	0.25–36 h
10	1.86	C_10_H_13_N_5_O_4_	268.1040 (−0.3)	266.0899 (+0.7)	136.0613 [M + H − ribose]^+^, 119.0347 [M + H − ribose − NH_3_]^+^	135.0315 [M − H − ribose]^−^, 107.0377 [M − H − C_6_ H_12_O_5_ − HCN]^−^	Adenine nucleoside ^a^	+	+	0.25–36 h
11	2.60	C_9_H_11_NO_2_	166.0864 (+1.0)	164.0717 (−0.12)	149.0588 [M + H − NH_3_]^+^, 131.0507 [M + H − NH_3_ − H_2_O]^+^, 120.0814 [M + H − CH_2_O_2_]^+^, 103.0550 [M + H − NH_3_ − CH_2_O_2_]^+^	147.0460 [M − H − NH_3_]^−^, 103.0571 [M − H − NH_3_ − CO_2_]^−^,	Phenylalanine ^a^	+	+	0.25–36 h
12	3.70	C_7_H_6_O_4_	-	153.0196 (+2.0)	-	109.0293 [M − H − CO_2_]^−^, 81.0349	Protocatechuic acid ^a^	+	+	0.25–36 h
13	4.01	C_18_H_26_O_12_	-	433.1355 (+0.9)	457.1319 [M + Na]^+^ (+0.25)	301.0939 [M − H − C_5_H_8_O_4_]^−^, 139.0399 [M − H − C_5_H_8_O_4_ − glc]^−^, 161.0454, 124.0168	Markhamioside F	+	-	0.25–8 h
14	4.87	C_11_H_12_N_2_O_2_	205.0970 (−0.25)	203.0830 (+2)	188.0704 [M + H − NH_3_]^+^, 170.0596 [M + H − NH_3_ − H_2_O]^+^, 159.0915 [M + H − CH_2_O_2_]^+^, 132.0806 [M + H − NH_3_ − CH_2_O_2_]^+^, 146.0600, 118.0652, 91.0553	185.0443 [M − H−NH_3_]^−^, 159.0916 [M − H−CO_2_]^−^, 142.0659 [M − H − NH_3_ − CO_2_]^−^, 116.0507, 74.0276	Tryptophan ^a^	+	+	0.25–36 h
15	5.34	C_7_H_6_O_3_	139.0388 (−0.8)	137.0249 (+3.6)	121.0278 [M + H − H_2_O]^+^, 111.0072 [M + H − CO]^+^, 95.0482 [M + H − CO_2_]^+^, 77.0400 [M + H − H_2_O − CO_2_]^+^	93.0369 [M − H − CO_2_]^−^, 75.0272 [M − H − H_2_O − CO_2_]^−^	Hydroxybenzoic acid ^a^	+	-	-
16	6.04	C_9_H_11_NO_3_	182.0810 (−0.6)	180.0666 (+0)	165.0550 [M + NH_3_]^+^	-	Tyrosine ^a^	+	+	0.25–36 h
17	8.17	C_16_H_18_O_9_	355.1024 (+0.3)	353.0878 (−0.11)	-	-	Chlorogenic acid ^a^	+	-	-
18	8.27	C_28_H_32_O_16_	625.1763 (0)	623.1624 (+1.1)	463.1225 [M + H − glc]^+^, 301.0707 [M + H − 2glc]^+^,	461.1109 [M − H − glc]^−^, 299.0574 [M − H − 2glc]^−^	Rhamnocitrin 3,4'-di-*O*-glucoside	+	-	0.25-6 h
19	8.97	C_9_H_8_O_4_	181.0490 (−2.8)	179.0357 (3.9)	153.0544 [M + H − CO]^+^, 137.0314 [M + H − CO_2_]^+^, 123.0051, 93.0364	135.0467 [M − H − CO_2_]^−^	Caffeic acid ^a^	+	-	0.25−0.5 h
20	9.49	C_9_H_10_O_5_	199.0600 (−0.4)	197.0455 (−0.14)	181.0478 [M + H − H_2_O]^+^, 155.0694 [M + H − CO_2_]^+^, 125.0228 [M + H − CO_2_ − CH_2_O]^+^, 97.0298, 77.0388	182.0231 [M − H − CH_3_]^−^, 166.9992 [M − H − CH_3_O]^−^, 153.0572 [M − H − CO_2_]^−^, 123.0098 [M − H − CO_2_ − CH_2_O]^−^, 95.0153	Syringic acid ^a^	+	+	0.25–36 h
21	9.95	C_29_H_38_O_16_	-	641.2085 (−0.28)	665.2056 [M + Na] ^+^ (+0.7)	479.1604 [M − H − glc]^−^, 317.1053 [M − H − 2glc]^−^, 195.0653 [M − H − 2glc − RDA]^−^, 121.0292	Isomucronulatol-2',5'-di-*O*-glucoside	+	-	0.25–3 h
22	10.19	C_27_H_30_O_15_	595.1652 (−0.8)	593.1512 (−1.1)	433.1137 [M + H − glc]^+^, 271.0596 [M + H − 2glc]^+^	-	Emodin-di-*O*-glucoside	+	-	0.25–4 h
23	10.40	C_9_H_10_O_4_	183.0654 (−0.8)	181.0511 (+2.6)	155.0696 [M + H − CO]^+^, 140.0466 [M + H − CO − CH_3_]^+^, 125.0220 [M + H − CO − CH_2_O]^+^, 123.0418 [M + H − 2CH_2_O]^+^, 95.0511, 77.0400	-	Syringaldehyde ^a^	+	-	0.25–6 h
24	10.73	C_17_H_24_O_9_	-	371.1352 (+1.3)	395.1314 [M + Na]^+^ (+0.4)	209.0824 [M − H − glc]^−^, 193.0506, 178.0269	Syringin ^a^	+	+	0.25–36 h
25	11.20	C_10_H_10_O_4_	195.0652 (+0.25)	193.0510 (+1.9)	177.0547 [M + H − H_2_O]^+^, 149.0590 [M + H − CH_2_O_2_]^+^, 163.0388 [M + H − CH_3_OH]^+^, 117.0333 [M + H − CH_2_O_2_ − CH_3_OH]^+^, 109.0271, 89.0380, 77.0387	178.0257 [M − H − CH_3_]^−^, 149.0611 [M − H − CO_2_]^−^, 134.0380 [M − H − CO_2_ − CH_3_]^−^	Ferulic acid ^a^	+	+	0.25–36 h
26	11.31	C_22_H_22_O_11_	463.1232 (−0.25)	461.1099 (+2.1)	301.0700 [M + H − glc]^+^, 269.0430 [M + H − glc − CH_3_OH]^+^, 241.0500, 213.0561, 197.0560	299.0568 [M − H − glc]^−^, 284.0336 [M − H − glc − CH_3_]^+^, 255.0308, 227.0334, 135.0092	Pratensein-7-*O*-glucoside	+	-	0.25–8 h
27	11.90	C_10_H_10_O_4_	195.0648 (−1.6)	193.0509 (+1.6)	177.0553 [M + H − H_2_O]^+^, 149.0631 [M + H − CH_2_O_2_]^+^, 117.0319 [M + H − CH_2_O_2_ − CH_3_OH]^+^, 89.0377	178.0259 [M − H − CH_3_]^−^, 149.069 [M − H − CO_2_]^−^, 134.0374 [M − H − CO_2_ − CH_3_]^−^	Isoferulic acid	+	+	0.25–36 h
28	12.03	C_11_H_12_O_5_	225.0759 (+0.8)	223.0617 (+2.3)	247.0578 [M + Na]^+^, 207.0648 [M + H − H_2_O]^+^, 179.0671 [M + H − CH_2_O_2_]^+^, 175.0409 [M + H−H_2_O − CH_3_OH]^+^, 147.0418 [M + H − CH_2_O_2_ − CH_3_OH]^+^	205.0514 [M − H − H_2_O]^−^, 179.0710 [M − H − CO_2_]^−^, 163.0401 [M − H − 2CH_2_O]^−^, 161.0608 [M − H − H_2_O − CO_2_]^−^, 133.0661, 117.0720, 91.0569	Sinapic acid	+	-	0.25–3 h
29	12.85	C_22_H_22_O_10_	447.1290 (+1.1)	445.1142 (+0.6)	469.1106 [M + Na]^+^ (−0.3), 285.0767 [M + H − glc]^+^, 270.0523 [M + H − glc − CH_3_]^+^, 253.0501, 225.0553, 137.0238	283.0620 [M − H − glc]^−^, 268.0366 [M − H − glc − CH_3_]^−^, 233.0890, 203.0819, 159.0361	Calycosin-7-*O*-glucoside ^a^	+	-	0.25–24 h
30	13.25	C_21_H_20_O_10_	433.1132 (+0.7)	431.0985 (+0.25)	271.0602 [M + H − glc]^+^, 215.0702	269.0390 [M − H − glc]^−^, 195.0509	Cosmosiin ^a^	+	+	0.25–36 h
31	15.08	C_29_H_38_O_15_	-	625.2156 (+2.9)	649.2115 [M + Na]^+^ (2.0), 487.1555 [M + Na − glc]^+^, 325.1390 [M + Na − 2glc]^+^, 177.0552	463.1635 [M − H − glc], 301.1097 [M − H − 2glc]^−^, 286.0850, 121.0300	Isomucronulatol-7,2'-di-*O*-glucoside	+	-	0.25–12 h
32	15.27	C_9_H_16_O_4_	189.1120 (−0.7)	187.0985 (+4.7)	211.0939 [M + Na]^+^ (+0.4), 165.1231	169.0875 [M − H − H_2_O]^−^, 143.1091 [M − H − CO_2_]^−^, 125.0980 [M − H − H_2_O − CO_2_]^−^, 97.0680, 95.0521	Azelaic acid	+	+	0.25–36 h
33	15.75	C_22_H_22_O_9_	431.1344 (+1.7)	429.1193 (+0.6)	453.1159 [M + Na]^+^ (+0.8), 269.0823 [M + H − glc]^+^, 254.0588, 213.0912	267.0681 [M − H − glc]^−^	Ononin ^a^	+	-	0.25–24 h
34	15.94	C_23_H_26_O_10_	463.1603 (+1.0)	461.1449 (−0.9)	301.1083 [M + H − glc]^+^,	167.0706	9,10-dimethoxypterocarpan-3-*O*-glucoside	+	-	0.25–12 h
35	16.83	C_9_H_14_O_2_	155.1065 (−1.1)	153.0923 (+1.4)	137.0969 [M + H − H_2_O]^+^, 109.1014 [M + H − CH_2_O_2_]^+^, 95.0859, 67.0569	-	2,4-Nonadienic acid	+	+	0.25–36 h
36	17.18	C_23 h28_O_10_	465.1754 (−0.3)	463.1611 (+0.21)	487.1574 [M + Na]^+^ (+0), 303.1225 [M + H − glc]^+^, 167.0704, 123.0439	301.1092 [M − H − glc]^−^, 271.0629 [M − H − glc − CH_3_O]^−^, 165.0489, 121.0307,	Isomucronulatol-7-*O*-glucoside	+	-	0.25–12 h
37	17.42	C_16_H_12_O_5_	285.0759 (+0.8)	283.0622 (+3.8)	270.0525 [M + H − CH_3_]^+^, 253.0490, 225.0534, 197.0596, 157.0641, 137.0223	239.0350, 211.0398, 195.0452, 167.0506, 148.0168, 135.0089	Calycosin ^a^	+	-	0.25–24 h
38	19.01	C_24_H_24_O_10_	473.1442 (−0.1)	-	455.3400 [M + H − H_2_O]^+^, 269.0795 [M + H−glc − C_2_H_4_O_2_]^+^	-	6'-*O*-acetyl ononin	+	-	0.25–8 h
39	20.09	C_16_H_12_O_4_	269.0809 (+0.25)	267.0669 (+2.4)	253.0503, 237.0552, 225.0551, 213.0918, 197.0599, 181.0647	251.0358, 223.0401, 195.0453, 167.0505, 132.0223	Formononetin ^a^	+	-	0.25–24 h
40	22.25	C_47_H_78_O_19_	947.5211 (+0.1)	945.5112 (+5.0)	969.5027 [M + Na]^+^ (−0.2)	783.4625 [M − H − glc]^−^, 654.4278, 541.1990	Astragaloside V	+	-	0.25–24 h
41	22.72	C_47_H_78_O_19_	947.5223 (+1.3)	945.5108 (+4.6)	749.4493,617.4075, 587.3937, 969.5037 [M + Na]^+^ (+0.8), 473.3647,455.3527, 437.3421, 305.1585, 143.1067, 125.0987	783.4651 [M − H − glc]^−^, 489.3570, 161.0466	Astragaloside VI	+	-	0.25–24 h
42	22.94	C_41_H_68_O_14_	785.4676 (−0.7)	783.4563 (3.5)	807.4505 [M + Na]^+^ (+0.25) 605.4145, 455.3490, 437.1977, 419.3328	621.4056, 489.3691, 161.0458	Astragaloside III	+	-	0.25–12 h
43	23.06	C_41_H_70_O_14_	-	785.4704 (+1.5)	809.4659 [M + Na]^+^ (+0.2)	623.4223 [M − H − glc], 491.3752 [M − H − glc − xyl]^−^, 415.3217, 616.0448	Cyclocanthoside E	+	-	3 h
44	24.03	C_47_H_78_O_19_	947.5216 (+0.6)	945.5116 (+5.5)	965.5039 [M + Na]^+^ (+1.0)	783.4630 [M − H − glc],765.4541, 651.4267, 489.3623, 179.0557, 161.0444	Astragaloside VII	+	-	0.25–12 h
45	24.41	C_41_H_68_O_14_	785.4682 (+0)	783.4569 (+4.2)	807.4089 [M + Na]^+^ (+0.9)	621.4019, 489.3605, 383.2991, 161.0453	Astragaloside IV ^a^	+	-	0.25–24 h
46	24.63	C_43_H_70_O_15_	827.4795 (+1.0)	825.4669 (3.3)	849.4615 [M + Na]^+^ (+1.0), 665.4299,647.4172, 473.3602, 455.3547, 437.3413, 175.0601	-	Astragaloside II	+	-	0.25−0.5 h, 6 h
47	24.89	C_48_H_78_O_18_	943.5254 (−0.7)	941.5163 (+5.1)	965.5072 [M + Na]^+^ (−0.8), 797.4635, 635.4105, 617.4020, 599.3916, 441.3704, 423.3577, 405.3532, 309.1134, 203.1766	923.5120, 733.4538, 615.4003, 457.3725, 247.0823, 205.7828	Soyasaponin I	+	-	0.25–24 h
48	24.97	C_47_H_76_O_17_	-	911.5032 (+2.5)	935.4967 [M + Na]^+^ (−0.8), 477.115	703.4545, 615.3994, 571.4053, 457.3800, 247.0808, 205.0718	Astragaloside VIII	+	-	0.25–24 h
49	25.71	C_45_H_72_O_16_	869.4898 (+0.6)	867.4785 (+4.4)	831.4571, 711.4038, 651.3955, 591.3675, 447.3377	765.4573	Astragaloside I	+	-	0.25−0.5 h
50	26.01	C_45_H_72_O_16_	869.4887 (−0.6)	867.4756 (+1.0)	711.4037, 477.3364	807.4652, 765.4568, 179.0567	Isoastragaloside I	+	-	3 h
51	27.35	C_47_H_74_O_17_	911.4998 (+0)	909.4888 (+3.8)	875.4590, 753.4225, 731.4360, 477.3428, 437.3368, 259.0854,	867.4787, 807.4666, 747.4616	Acetyl astragaloside I	+	-	8 h
M1	14.34	C16 H_12_O_8_S	365.0323 (−0.5)	363.0177 (−0.8)	285.0782, 270.0522, 225.0537, 137.0254	283.0609, 268.0375, 211.0407, 148.0174	Sulfated calycosin	-	-	0.25–8 h
M2	14.82	C_22_H_20_O_11_	461.1081 (+0.7)	459.0928 (−0.9)	285.0766, 270.0534, 225.0541, 137.0230	283.0617, 268.0385, 239.0346, 211.0408, 175.0255, 117.0191, 113.0254, 85.0320	Glucuronidated calycosin	-	-	0.25–12 h
M3	15.41	C_22_H_20_O_10_	445.1133 (+1.0)	443.0979 (−0.9)	269.0817, 253.0486, 237.0516, 213.0904, 181.0650	267.0665, 252.0441, 175.0243, 113.0249	Glucuronidated formononetin	-	-	0.25–8 h
M4	16.09	C_22_H_20_O_12_	477.1027 (−0.1)	475.0883 (+0.3)	301.0761, 286.0475, 167.0661	299.0583, 284.0328, 255.0291, 227.0344, 175.0242, 151.0391, 113.0261	Glucuronidated rhamnocitrin	-	-	0.25–8 h
M5	16.28	C_17_H_18_O_8_S	383.0798 (+0.9)	381.0649 (+0.1)	-	301.1082, 286.0827, 271.0624, 256.0363, 135.0450, 109.0312	Sulfated isomucronulatol	-	-	0.25–8 h
M6	16.85	C_16_H_12_O_7_S	349.0378 (+0.6)	347.0228 (−0.6)	269.0792, 253.0507, 213.0941, 181.0629	267.0667, 252.0429, 223.0390	Sulfated formononetin	-	-	0.25–8 h
M7	17.27	C_23_H_26_O_11_	479.1544 (−0.7)	477.1396 (−1.2)	303.1219, 167.0707	301.1085, 286.0855, 271.0615, 256.0370, 175.0252, 135.0449, 113.0254	Glucuronidated isomucronulatol	-	-	0.25–12 h
M8	18.45	C_16_H_12_O_6_	301.0709 (+0.8)	299.0561 (+0.3)	-	284.0305, 255.0312, 227.0353, 183.0432, 135.0085	Rhamnocitrin	-	-	0.25–8 h
M9	19.63	C_17_H_18_O_5_	303.1229 (+0.7)	301.1084 (+1.0)	181.0841, 167.0688, 135.0339, 123.0440	286.0836, 271.0599, 256.0377, 135.0445, 121.0245, 109.0340	Isomucronulatol	-	-	0.25–8 h

^a^ Compounds identified in comparison with authentic standards; RA: Radix Astragali; dosed plasma: 0.25, 0.5, 1, 1.5, 2, 3, 4, 6, 8, 12, 24 and 36 h; M: the metabolites identified in plasma.

### 2.2. Characterization of the Absorbed Constituents in Mice Plasma

The peaks that appeared at the same position in the total ion chromatograms of both the dosed mice bio-samples and the RA extract, but not in the chromatograms of the controlled mice bio-samples, were regarded as absorbed constituents [22].

As a result, 31 compounds, shown in Table 1, were detected as the absorbed constituents in immunosuppressive mice, including disaccharide (Peak 3), raffinose (Peak 6), nicotinic acid (Peak 8), markhamioside F (Peak 13), rhamnocitrin-3,4'-di-*O*-glucoside (Peak 18), caffeic acid (Peak 19), isomucronulatol-2',5'-di-*O*-glucoside (Peak 21), emodin-di-*O*-glucoside (Peak 22), syringaldehyde (Peak 23), pratensein-7-*O*-glucoside (Peak 26), sinapic acid (Peak 28), calycosin-7-*O*-glucoside (Peak 29), isomucronulatol-7,2'-di-*O*-glucoside (Peak 31), ononin (Peak 33), 9,10-dimethoxypterocarpan-3-*O*-glucoside (Peak 34), isomucronulatol-7-*O*-glucoside (Peak 36), calycosin (Peak 37), 6'-*O*-acetyl ononin (Peak 38), formononetin (Peak 39), astragaloside V (Peak 40), astragaloside VI (Peak 41), astragaloside III (Peak 42), cyclocanthoside E (Peak 43), astragaloside VII (Peak 44), astragaloside IV (Peak 45), astragaloside II (Peak 46), soyasaponin I (Peak 47), astragaloside VIII (Peak 48), astragaloside I (Peak 49), isoastragaloside I (Peak 50) and acetyl astragaloside I (Peak 51).

Of these absorbed constituents, 20 compounds were discovered at 0.25 h and disappeared from 6 to 24 h after RA oral administration. Peaks 3, 6, 8, 19, 21, 22, 28 and 49 appeared within 4 h, indicating a faster metabolism. Peaks 43, 50 and 51 were only observed at 3, 3 and 8 h, respectively, significantly slower than other saponins. This indicated that Peaks 43, 50 and 51 might be the metabolites produced by other saponins.

### 2.3. Identification of Metabolites in Mice Plasma

**Figure 3:**
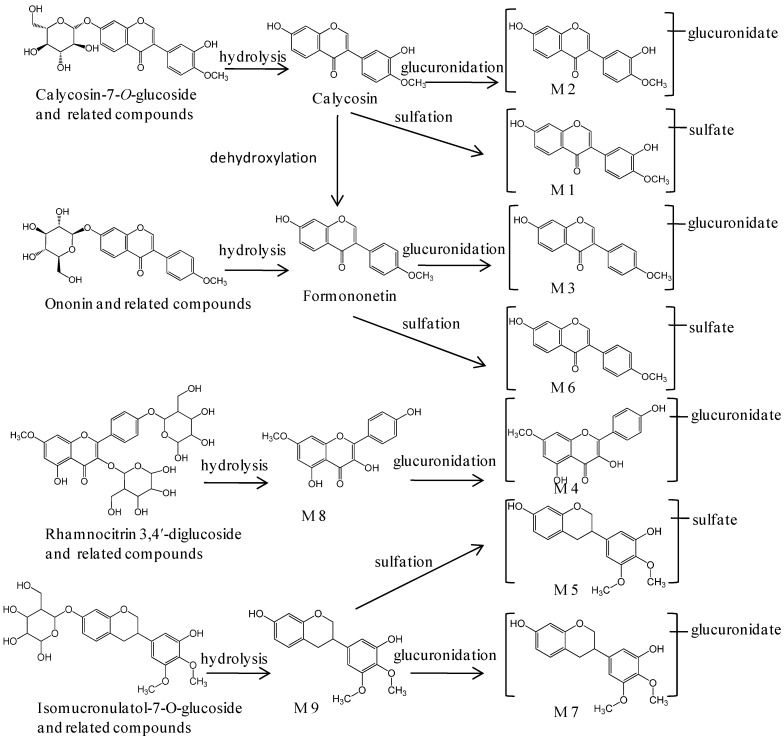
The possible metabolic pathways of nine metabolites in Balb/c mice orally administered with RA.

Metabolites identification was carried out according to the drug major metabolic pathways and metabolic reactions types, such as oxidation, deduction, hydrolyzation, glucuronidation and sulfation [23]. As shown in Table 1, nine metabolites were originated from flavonoids with detailed information, including the retention time, chemical composition, molecular weight and characteristic fragment ions. They were highly associated with their likely original compounds and identified according to the metabolism and metabolic pathway of flavonoids *in vivo* [24,25]. Metabolites M1, M3, M4, M5, M6, M8 and M9 were present in plasma samples from 0.25 to 8 h, while M2 and M7 were last, till 12 h. In addition, the metabolic pathways of these metabolites were derived as shown in Figure 3.

Metabolite M1, with a retention time of 14.34 min, showed a precursor ion at *m*/*z* 365.0323 (C_16_H_12_O_8_S). The neutral loss of 80 Da from the protonated parent ion gave the major fragment ion at *m*/*z* 285, indicating the presence of a sulfate group (SO_3_, 80 Da). The other product ions were similar to that of calycosin. Metabolite M2 had a retention time of 14.82 min and a protonated molecule at *m*/*z* 461.1081 (C_22_H_20_O_11_), 176 Da higher than calycosin. The neutral loss of the glucuronic acid group made the fragment ion at *m*/*z* 285, and the characteristic fragment ions at *m*/*z* 270, 225 and 137 were observed. Thus, M1 and M2 were proposed to be sulfated calycosin and glucuronidated calycosin, respectively.

Metabolite M3 and M6 showed precursor ions at *m*/*z* 445.1133 (C_22_H_20_O_10_) and 349.0378 (C_16_H_12_O_7_S), 176 and 80 Da higher than formononetin, respectively. Then, they were identified as glucuronidated formononetin and sulfated formononetin, respectively, due to their characteristic fragment ions at *m*/*z* 267, 253, 213 and 181, being similar to those of formononetin.

Metabolite M8, which was eluted at 18.45 min, showed a precursor ion at *m*/*z* 301.0709 in positive ion mode and *m*/*z* 299.0561 in negative ion mode, which demonstrated the chemical composition of C_16_H_12_O_6_. Metabolite M4, with a retention time of 16.09 min, gave a deprotonated ion at *m*/*z* 475.0883 (C_22_H_20_O_12_), 176 Da more than M8, and the fragment ions at 284, 255 and 227 suggested that M4 and M8 have the same aglycone. Therefore, M4 and M8 were characterized as glucuronidated rhamnocitrin and rhamnocitrin, respectively.

Metabolite M9, with a retention time of 19.63, showed a protonated ion at *m*/*z* 303.1229, indicating an element composition of C_17_H_18_O_5_. M5 was eluted at 16.28 min, with a protonated molecular weight of 383.0798 Da (C_17_H_18_O_8_S), which was 80 Da higher than M9. M7 was eluted at 17.27 min, and a chemical composition of C_23_H_26_O_11_ was suggested by the accurate mass measurement. The product ion at *m*/*z* 303.1219 was formed by the neutral loss of 176 Da, suggesting that M7 was the glucuronidated product of M9. Thus, M5, M7 and M9 were determined to be sulfated isomucronulatol, glucuronidated isomucronulatol and isomucronulatol, respectively.

### 2.4. Pharmacokinetics of Calycosin-7-O-glucoside, Ononin, Calycosin, Formononetin and Astragaloside IV in Mice Plasma

#### 2.4.1. Assay Validation

The data for method validation are shown in Table 2. There was no impurity that interfered with the determination of five target compounds by testing six sources of blank plasma. The linearity showed a good relationship with the correlation coefficient *r* from 0.9923 to 0.9994. The intra-day and inter-day precision were assessed by QC samples at three concentration levels, and the results indicated that the bias was within acceptable limits; the relative standard deviation (RSD) was from 1.22% to 4.37%. The accuracy was from 95.21% to 104.58%, with the RSD less than 3.69%. These results indicated that the developed HPLC-MS/MS method was a reliable and useful method for quality assessment of calycosin-7-*O*-glucoside, ononin, calycosin, formononetin and astragaloside IV in mice plasma.

**Table 2:** Method validation for the determination of calycosin-7-*O*-glucoside, ononin, calycosin, formononetin and astragaloside IV.

Compounds	Linear Range (ng/mL)	Linearity	*r*	LOD (ng/mL)	LOQ (ng/mL)	Intra-Day RSD (*n =* 6)	Inter-Day RSD (*n =* 3)	Accuracy	
								Mean (%)	RSD (%)
Calycosin-7-*O*-glucoside	4.48–448.00	*y* = 0.7300*x* − 0.0017	0.9982	0.64	2.28	1.28	3.20	95.21	1.63
Ononin	3.40–340.00	*y* = 0.2847*x* + 0.0009	0.9994	0.52	1.70	2.16	3.11	97.03	2.18
Calycosin	4.47–447.00	*y* = 0.7951*x* − 0.0047	0.9987	0.84	2.24	1.22	2.95	104.58	2.56
Formononetin	5.52–552.00	*y* = 0.3547*x* + 0.0021	0.9949	0.68	2.76	2.77	3.64	102.21	1.40
Astragaloside IV	8.98–898.00	*y* = 0.8558*x* + 0.0031	0.9923	1.28	4.49	3.84	4.37	98.48	2.87

RSD: relative standard deviation.

#### 2.4.2. Pharmacokinetics Study

**Figure 4:**
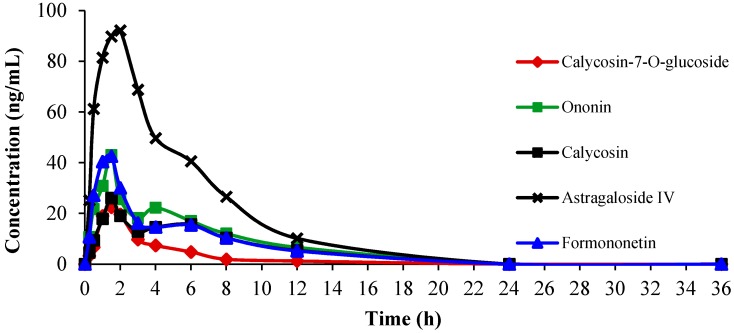
The mean plasma concentration-time profiles pf calycosin-7-*O*-glucoside, ononin, calycosin, formononetin and astragaloside IV in immunosuppressive mice plasma after oral administration of RA.

The pharmacokinetic parameters of the area under the concentration-time curve from 0 to 36 h (AUC_0–36 h_) and the half-life time (*t*_1/2_) were estimated using a statistical moment theory by the Drug and Statistics 2.0 software (DAS, version 2.0, the Mathematical Pharmacology Professional Committee of China, Shanghai, China). The maximum plasma concentration (*C*_max_) and the time of maximum plasma concentration (*t*_max_) were obtained from the observed data (Table 3). The plasma concentration-time profiles for calycosin-7-*O*-glucoside, ononin, calycosin, formononetin and astragaloside IV are shown in Figure 4. The *t*_max_ of calycosin-7-*O*-glucoside, ononin and calycosin was the same at 1.5 h, and no significant differences of t_1/2_ was observed. In the four isoflavonoids, formononetin showed relatively slow absorption and elimination with *t*_max_ at 2 h and *t*_1/2_ at 3.99 h. Meanwhile, ononin, calycosin and formononetin displayed a double-peak phenomenon after oral administration of RA, appearing at 4, 6 and 6 h, respectively. Astragaloside IV, as one saponin, showed relatively more absorption and slower excretion reflected by the data of AUC_0–*t*_ 695.37 ± 178.57 μg/L∙h, *C*_max_ 128.95 μg/L and *t*_1/2_ 3.48 ± 1.15 h.

**Table 3:** The pharmacokinetic parameter of calycosin-7-*O*-glucoside, ononin, calycosin, formononetin and astragaloside IV.

Compounds	Parameter				
	AUC_(0–*t*)_ (ng/mL∙h)	AUC_(0–∞)_ (ng/mL∙h)	*t*_1/2_z (h)	*t*_max_ (h)	*C*_max_ (ng/mL)
Calycosin-7-*O*-glucoside	108.16 ± 37.14	127.17 ± 40.31	2.61 ± 0.67	1.5	33.41
Ononin	227.84 ± 59.69	239.24 ± 47.38	1.96 ± 0.34	1.5	51.38
Calycosin	135.88 ± 41.41	154.87 ± 37.69	2.21 ± 0.92	1.5	32.98
Formononetin	233.38 ± 47.83	256.42 ± 45.68	3.99 ± 1.03	2.0	47.93
Astragaloside IV	695.37 ± 178.57	722.49 ± 196.52	3.48 ± 1.15	1.5	128.95

### 2.5. *Discussion*

The illustration of the chemical basis for the therapeutic effect has always been an important part of the research on TCMs, due to their varied constituents having different targets. It was thought that compounds absorbed into the blood had a chance to show bioactivities. Therefore, pharmacokinetic profiles of the absorbed components of RA were necessary for confirming the effective compounds *in vivo*. In addition, after oral administration of TCMs, the concentrations of some compounds are quite low, increasing the complexity of their detection in biological samples. In recent decades, researchers have developed some methods to clarify the chemical basis for TCMs, such as fingerprint chromatography [26], serum pharmacology [27] and serum pharmacochemistry [28]. In the present research, serum pharmacochemistry was used based on our previous studies [14,18,20], trying to profile the potentially active constituents in RA by the ultra-fast liquid chromatography (UFLC)-DAD-Q-TOF-MS/MS technique.

Flavonoids and saponins as the two main kinds of constituents were detected in plasma after the immunosuppressive mice were administrated RA orally. Among the absorbed constituents, 11 flavonoids and 12 triterpenoid saponins accounted for almost three-quarters of the 31 compounds. As shown in Figure 2 and Table 1, the flavonoids and saponins were found to have relatively high response values, relatively faster absorption and relatively long retention times, indicating that flavonoids and saponins might play an important role in the therapeutic effect. Moreover, a number of research works have been performed to confirm the relationship between flavonoids and saponins in RA and the immunomodulatory effect. Zheng *et al.* [29] provided different lines of evidence that the RA flavonoids stimulated the expression of erythropoietin in cultured human embryonic kidney fibroblasts *in vitro*. Two isoflavonoids, namely calycosin and calycosin-7-*O*-glucoside, showed an inhibition effect on high glucose-induced mesangial cell early proliferation, indicating the significant therapeutic potential to modulate the development and/or progression of diabetic nephropathy [30]. Meanwhile, calycosin could trigger cell apoptosis through the mitochondrial apoptotic pathway by upregulating RASD1 in human breast cancer cells, MCF-7 [31]. Astragaloside IV was one of the most common saponin compounds in RA with a variety of biological activities, including ameliorating renal fibrosis by blocking the TGF-β/Smad signaling pathway *in vivo* and *in vitro* [32], preventing damage to human mesangial cells [33], preventing the development of chronic asthma activities [34], attenuating allergic inflammation by regulationTh1/Th2 cytokine and enhancing CD4^+^CD25^+^Foxp3 T-cells in ovalbumin-induced asthma [8]. All effects of flavonoids and saponins in RA were closely related to the immune system, indicating that they contributed to immune regulation in the chemotherapy-induced immunosuppressive condition. This study provides helpful chemical information for further understanding the pharmacological and active mechanism research on RA.

In the present study, five major components were determined in mice plasma for the pharmacokinetic study by the HPLC-MS/MS method. The double-peak phenomenon as a common phenomenon in the absorption of flavonoids was found, possibly resulting from enterohepatic circulation, transformation of different compounds, double-site absorption and intestinal efflux [35]. The first absorption peak may be beneficial to produce effects in a timely manner, and the second absorption peak may help to maintain the effects. In previous reports, no t_1/2β_ was calculated for calycosin-7-*O*-glucoside, ononin and calycosin and formononetin, because of the rapid elimination [36]. Moreover, in the formula, calycosin-7-*O*-glucoside, ononin, calycosin and formononetin appeared together in a single and plateau absorption phase, and astragaloside IV presented the phenomenon of double peaks, with the *t*_max_ less than 0.75 h [37]. The differences in the pharmacokinetic features might be a result of the difference of the pathological conditions between model animals and normal animals and/or the complex composition of herbal medicine.

The nine metabolites of flavonoids detected in mice plasma were useful for better understanding the pharmacological mechanism and the clinical application. Glucuronidation and sulfation are the principal metabolic pathways of flavonoids in phase II metabolism [38,39]. In this study, 4-glucuronidated- and 3-sulfated-flavonoids were tentatively identified, which might affect the bioavailability of their aglycone. In addition, the metabolites of saponins could not be detected obviously in mice plasma. These pharmacokinetic profiles of multiple absorbed components and their metabolites might be beneficial to the overall comprehensive effects of the oral administration of RA on immunosuppressive disease.

## 3. Experimental Section

### 3.1. Chemicals and Reagents

The authentic standards of asparagine, choline, γ-aminobutyric acid, raffinose, adenine, nicotinic acid, leucine, adenine nucleoside, phenylalanine, protocatechuic acid, tryptophan, hydroxybenzoic acid, tyrosine, chlorogenic acid, syringic acid, syringaldehyde, syringin, ferulic acid, cosmosiin, formononetin, calycosin, astragaloside IV and naringenin (internal standard, IS) were purchased from the National Institute for the Control of Pharmaceutical and Biological Products (Beijing, China). Calycosin-7-*O*-glucoside and ononin were from Sigma-Aldrich (St. Louis, MO, USA). Astragaloside I and astragaloside III were isolated previously from RA herb in our laboratory by a Shimadzu LC-6AD system on Ultimate XB C18 (4.6 mm × 250 mm, 5 μm) and were unequivocally confirmed by spectroscopic methods (^1^H-NMR and ^13^C-NMR). All standards were detected with a purity of more than 95% by the area normalization method using HPLC and then prepared in methanol to a concentration of approximately 10 μg/mL for use.

Cyclophosphamide was from Shanxi Pude Pharmaceutical Co., Ltd. (Datong, China), and then dissolved in sterilized physiological saline prior to injection into Balb/c mice.

Throughout the entire experiment, methanol of HPLC grade (Fisher Scientific, Pittsburgh, PA, USA) and distilled water purified by a Milli-Q system (Millipore, Milford, MA, USA) were used. Other reagents were of analytical grade from Tianjin Fuyu Chemical Co., Ltd. (Tianjin, China).

### 3.2. Plant Material and Sample Preparation

RA is derived from the root of *Astragalus membranaceus* (Fisch.) Bge. var. mongholicus (Bge.) Hsiao, which was purchased from RA GAP base (Hunyuan, China). The species was identified by Wenbo Liao from Sun Yat-sen University, and a voucher specimen was deposited in our laboratory.

The extract of RA was prepared by following methods: roughly weighed RA (300 g) was boiled in 10 times the amount of water for 1 h; this extraction was repeated thrice; after combining the filtrates, the total extract was concentrated using a rotary evaporator (EYELA N-1001, Tokyo, Japan) at 70 °C; finally, 100 mL of a brown, concentrated solution (equal to 3.0 g raw material/mL) were obtained and then stored at 4 °C before being used.

### 3.3. Animals and Experiment Design

Male Balb/c mice (18–20 g body weight) were purchased from the Experimental Animal Center of Southern Medical University (Guangzhou, China) and kept in 12 h dark/light cycle room at a temperature of 21 ± 5 °C and a relative humidity of 55% ± 10% for at least a week before use. Animals were fed on standard laboratory diet and water *at libitum*. All experimental procedures were conducted in accordance with the guidelines for the Animal Care and Use Committee of Southern Medical University (Guangzhou, China).

The animal model of cyclophosphamide-induced immunosuppression in Balb/c mice in the present experiment has been published in previous reports [13,20]. Following randomization into study groups, mice (6/group/male) received intraperitoneal injection once daily of cyclophosphamide (i.p. 80 mg/kg BW) for 6 days. Additionally, the control group was injected intraperitoneally with sterile physiological saline. Blood samples (0.2 mL) were collect using spare sampling after dosing of 6 g/kg BW at 0.5, 2 and 6 h from Group I, 1, 3, 8 and 24 h from Group II, and 1.5, 4, 12 and 36 h from Group III. Plasma was harvested by centrifugation at 3000× *g* for 10 min and then kept frozen at −70 °C until analysis.

### 3.4. Preparation of Plasma Samples

To confirm the bioactive compounds *in vivo*, an aliquot of 120 μL of mixed plasma (20 μL from each mouse) was precipitated with 600 μL acetonitrile. After being vortexed for 1 min, the sample was centrifuged at 13,000× *g* at 4 °C for 30 min. The supernate was transferred into a fresh tube and evaporated with nitrogen. Then, the residue was dissolved in a 60-μL mobile phase with vortex mixing for 3 min and centrifuged at 13,000× *g* for 30 min. Finally, 10 μL of the supernatant were injected into the UFLC/DAD/Q-TOF-MS/MS system.

To quantify calycosin-7-*O*-glucoside, ononin, calycosin, formononetin and astragaloside IV in mice, a 10-μL IS solution was added into 50 μL of plasma and mixed for 30 s. Then, 1 mL ethyl acetate was added to the tube and vortexed for 3 min. After the sample was centrifuged at 13,000× *g* for 10 min, the upper organic layer was transferred into a fresh tube and evaporated with nitrogen. Finally, the residue was dissolved in a 50-μL mobile phase with vortex-mixing for 3 min and centrifuged at 13,000× *g* for 30 min. Ten microliters of the supernatant were injected into the HPLC-MS/MS system.

### 3.5. Bioanalytical Methods

The multiple constituents in RA and the absorbed compounds in mice plasma were identified by the ultra-fast liquid chromatography/diode-array detector/quadrupole time-of-flight tandem mass spectrometry (UFLC/DAD/Q-TOF-MS/MS) method. The chromatography and MS/MS conditions for identification were previously published [18].

The quantification was performed by an LC-MS/MS system, which consisted of an Agilent 1200 HPLC series system and an Agilent 6410 triple quadrupole mass spectrometer (Agilent Technol., Santa Clara, CA, USA) equipped with an electrospray ionization source. Samples were separated by a rapid resolution HT C18 column (2.1 mm × 50 mm, 1.8 μm, Agilent Technol.) at 30 °C. The mobile phase consisted of 0.1 formic acid both in methanol (A) and water (B) using a gradient elution program of 25%–50% A (0–5 min) and 50%–90% (5–10 min). The flow rate was 0.2 mL/min, and the injection volume was 10 μL. Detection was performed in the positive ion mode using multiple reaction monitoring (MRM) of the *m*/*z* 447.1/285.0 for calycosin-7-*O*-glucoside, *m*/*z* 431.1/269.0 for ononin, *m*/*z* 285.0/137.0 for calycosin, *m*/*z* 269.0/213.0 for formononetin, *m*/*z* 807.4/627.3 for astragaloside IV and *m*/*z* 271.1/151.0 for naringenin.

The method validation was executed in according with the principles of ICH Guideline Q2 with specificity, accuracy, linearity, precision, detection limit (LOD) and quantitation limit (LOQ) [40]. The linearity was determined on six serial working solutions by plotting the peak areas *versus* their concentrations, and the ranges were 4.48–448.00, 3.40–340.00, 4.47–447.00, 5.52–552.00 and 3.98–398.00 ng/mL for calycosin-7-*O*-glucoside, ononin, calycosin, formononetin and astragaloside IV, respectively. Quality control standards were prepared at three concentrations: one within 3 × the LOQ (low QC sample), one near the center (middle QC) and one near the upper boundary of the standard curve (high QC). Intra- and inter-day precision was performed by analyzing the standard mixture solution six times within a day and three times for three consecutive days. Accuracy was calculated as the percentage of recovery by the assay of the known added amount of reference standards in the sample. The LOD and LOQ were defined as the signal-to-noise ratio (*S*/*N*) of about 3 and 10, respectively.

## 4. Conclusions

In this work, we profiled the absorbed constituents after oral administration of RA in cyclophosphamide-induced immunosuppressive mice by serum pharmacochemistry with UFLC/DAD/Q-TOF-MS/MS. Based on chemical analysis, 31 of 51 absorbed constituents, including 11 flavonoids, 12 triterpenoid saponins, eight miscellaneous and nine metabolites, were detected in mice plasma. In addition, the pharmacokinetic properties of calycosin-7-*O*-glucoside, ononin, calycosin, formononetin and astragaloside IV indicated that their absorptions were relatively rapid. These results offered useful information for understanding the material bases of the therapeutic effects of RA and for its further development. However, this work only focused on describing the absorbed chemical constituents, and a new strategy is needed to reflect the internal relation and the integer synergism of multiple compounds. These problems are still under investigation.
